# Musculoskeletal symptoms among adult smartphone and tablet device users: a retrospective study

**DOI:** 10.1186/s40945-020-00096-6

**Published:** 2021-01-09

**Authors:** Ella Thorburn, Rodney Pope, Shaoyu Wang

**Affiliations:** School of Community Health, Charles Sturt University, Orange, NSW Australia

## Abstract

**Background:**

Previous literature suggests the use of smartphones and tablet devices may result in various postural and musculoskeletal disorders, predominantly of the neck and upper extremities. However, factors that contribute to the symptoms have not been adequately explored.

**Methods:**

This study aimed to investigate the prevalence and patterns (e.g. types, sites and temporal distributions) of musculoskeletal symptoms among adult smartphone and tablet device users. It also investigated device usage in terms of usage time, postures adopted during use, operational methods and purposes of device use in order to explain the symptom occurrences. Participants from eastern states of Australia retrospectively reported device usage and symptoms in the preceding two-week period, via an online survey. Data was analysed using Chi-square [χ2] analysis to determine the relationships between categorical variables; Mann-Whitney U tests to compare two groups (e.g. smartphone-only users versus tablet users) where dependant variables were ordinal; independent samples t-tests if dependent variables were continuous and approximated a normal distribution; and Spearman’s correlation analysis to assess the relationships between pairs of continuous or ordinal variables.

**Results:**

Of the 207 participants, 59.9% reported musculoskeletal symptoms during or after device use; for 64.5% of these, symptoms began within the first 30 min (mostly between 15 and 30 min) of commencing usage. No statistically significant differences were observed between smartphone-only users and tablet device users in proportions reporting symptoms during device use (χ2 = .350, *N* = 207, *p* = .554). The most prevalent symptom was stiffness. The most prevalent symptom occurred in the neck (18.1% in smartphone-only users and 19.3% in tablet device users). Tablet users who were 18–24 year-old and used their device for more than 30 min in each usage session more often experienced symptoms (82.4% prevalence) than those who used a device for 30 min or less (52.2%) (χ2 = 4.723, *N* = 63, *p* = .030).

**Conclusion:**

These findings suggest that user age, duration and frequency of usage, and type of device are important factors to consider in the formation of evidence-based guidelines to reduce experiences of musculoskeletal symptoms among smartphone and tablet device users. If usage was capped at < 15 min, the majority of smartphone and tablet device users would avoid symptoms.

## Introduction

Ownership of handheld devices, such as smartphones and tablet devices, is increasing exponentially [17, 20]. Smartphone and tablet device use penetrates all facets of life, enabling better standards of living through improved access to entertainment, more efficient education and work, and inclusion in health care [11, 14]. However, prolonged usage has been found to have negative impacts on physical health, predominantly of the neck and upper extremities [5, 10, 19]. A number of risk factors have been investigated for their association with musculoskeletal symptoms, including gender, posture, total time spent on a device, and types of tasks performed on devices [13, 22]. It is also suggested that relative importance of the risk factors may differ depending on specific populations.

Young et al. [23] demonstrated that larger displays and holding designs of mobile devices are associated with increased neck flexion and wrist extension. Given that smartphones and tablet devices differ in size, weight and manner of usage, their use may generate differing symptom patterns between users. However, there is minimal research on whether different smartphone and tablet devices cause distinctive postures and musculoskeletal symptoms [12, 22, 24]. Usage of these devices is likely to increase with continued technological developments. It is imperative to better understand musculoskeletal symptoms to establish guidelines for safe smartphone and tablet device use.

Within this context, the current research aimed to: 1) investigate the prevalence and patterns (including types, sites and temporal patterns) of musculoskeletal symptoms among smartphone and tablet device users; 2) investigate the usage of smartphones and tablet devices in terms of time, postures adopted during usage, operational methods and purposes of use (e.g. work); and 3) compare smartphone and tablet device users with regard to these variables. The study was designed to gather data on the maximum amount of time a smartphone or tablet device can be safely used prior to the onset of musculoskeletal symptoms, as well as how demographics and usage factors are associated with the symptoms.

## Method

### Study design

A survey design, using an online questionnaire, was employed, in which participants were asked to report on musculoskeletal symptoms and device usage within the preceding two-weeks. The term ‘musculoskeletal symptoms’ in this study included any physical symptoms in muscles, joints, bones and soft tissue, including but not limited to pain, stiffness, aches, discomfort, numbness and paraesthesia. Survey designs are commonly used for conducting research on this topic [22] and enable participants to record their symptoms and usage anonymously. The study was approved by the Charles Sturt University Human Research Ethics Committee (Protocol number: **H18271**).

### Participants and recruitment strategy

The survey population included Australian adults (over 18 years of age) residing in eastern states of Australia (Victoria, New South Wales, and Queensland; total population approximately 19.5 million at the time of the survey [3]), who used either a smartphone or tablet device. These states were included as it was assumed the device usage and network access within them would be comparable due to their shared networks, similar levels of coverage and access to devices. The survey was primarily advertised to populations in NSW, in areas including Western Sydney and the Greater West of NSW, to capture both metropolitan and regional experiences. However, being an online survey, the questionnaire was accessible to anyone, and responses from outside the eastern states were removed during data cleaning. The desired sample size for this study was at least 100 participants to ensure that population estimates based on the survey sample would be within approximately +/− 10% of the underlying population values, assuming a 95% confidence level and the large population of adults residing in the Australian eastern states (calculated using: https://www.surveymonkey.com/mp/sample-size-calculator/).

Participants were recruited through advertisements via radio, email and online. Specifically, advertisements were made through the Charles Sturt University (CSU) Facebook page and a CSU News article which received 967 unique views. Additional Facebook posts were made, sharing the News article to CSU student groups and personal pages of the researchers. Emails were sent to CSU research committees, physiotherapy staff and students at CSU, based on the researchers’ established networks. An interview was broadcast on the ABC radio station (Greater West) which directed people to the CSU News article. Participants in QLD and VIC were recruited only through Facebook.

Upon clicking on the survey link, prospective participants were first presented with an information sheet and consent statement to ensure they understood what was involved in participating in the survey and consented on that basis.

### Data collection procedure and tools

**Fig. 1:**
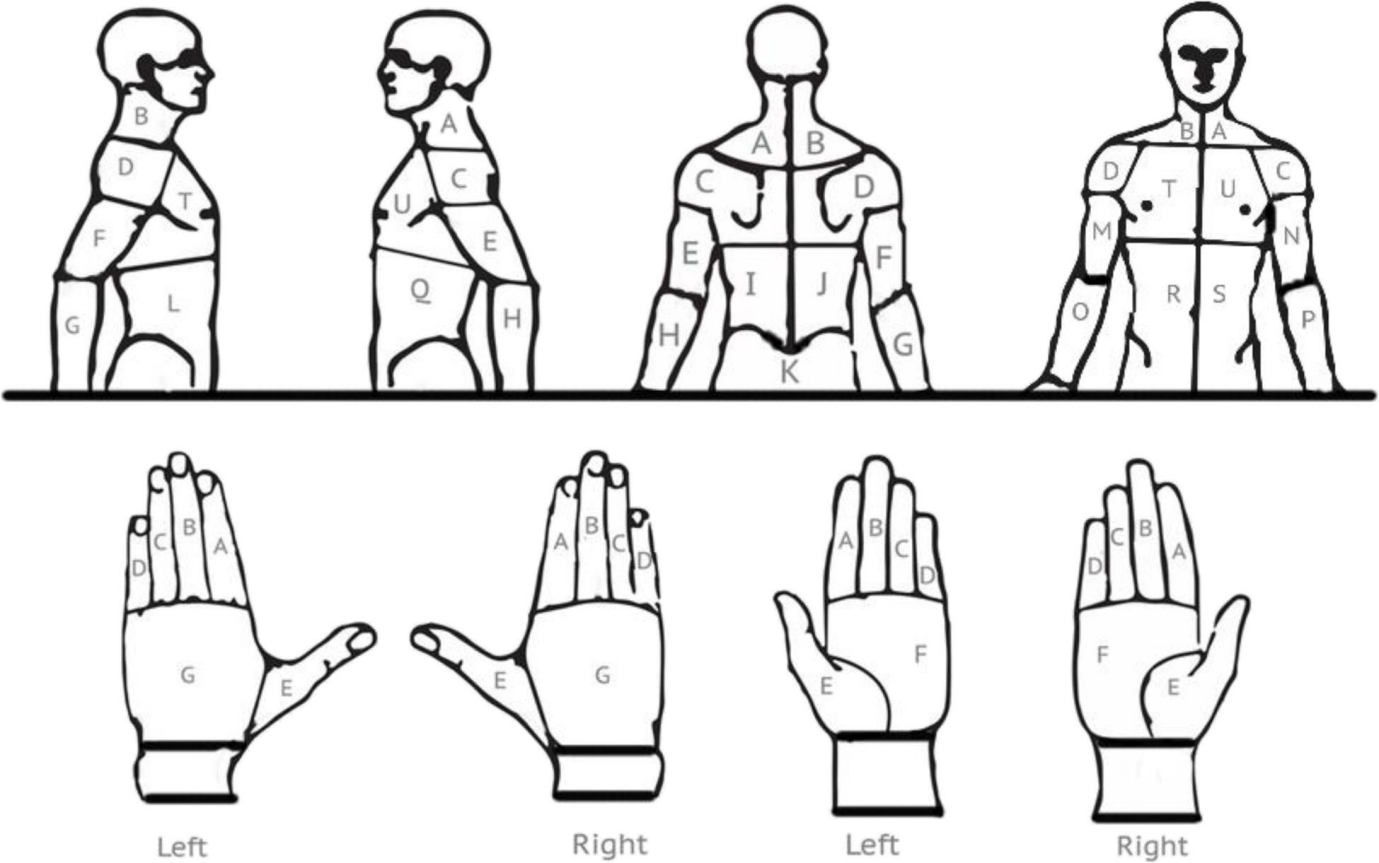
Body chart showing the individual regions on the questionnaire

All data were collected online via an anonymous questionnaire hosted through the online survey platform, SurveyMonkey. A copy of the survey used is available as a supplementary file. Participants were asked to answer questions regarding basic demographic information and their smartphone and tablet device use (including frequency and duration) over the two-week period prior to accessing the survey. Participants were asked about their experience of any musculoskeletal symptoms during this two-week period, such as pain and discomfort. Respondents were further asked to report the locations of their symptoms using a body chart (Fig. 1). Participants also reported body positions commonly adopted while using a device and how they held and operated the device.

Prior to administration, the questionnaire was reviewed by University academics and students to screen for common errors such as double barrelled, confusing or leading questions.

### Outcome measures

A visual analogue scale (VAS) was used within the questionnaire to rate pain on a scale of 0–10, with 10 indicating the worst pain and 0 indicating no pain. Olaogun et al. [15] showed strong intra-tester correlations between VAS of each tester (*p* < .05), and strong inter-tester correlations for VAS and for semantic differential scale (*p* < .05) on a musculoskeletal pain population. A body chart (Fig. 1) was created to improve the specificity in reporting of musculoskeletal symptoms. Although body charts have not been commonly used in this area of research, Xie et al. [22] found them to be a useful tool to support validity of key findings during analysis.

### Additional explanatory variables

The following additional variables, which may have contributed to explaining device use or musculoskeletal symptoms, were also recorded in the questionnaire: gender, age, height and weight used to compute body mass index (BMI), previous injuries, postural habits during device use, and reason for device use (work, education, leisure and other).

### Data analysis

Data were exported from SurveyMonkey to SPSS statistical analysis software (IBM SPSS version 25, 2017) for cleaning and analysis. Responses were excluded if they did not originate from eastern states, or if they were incomplete (> 3 demographic questions not answered or no response regarding symptom prevalence). Data were first analysed descriptively to provide an overview of demographic information, symptom prevalence and symptom patterns. The selection of parametric or non-parametric statistical tests was informed by visual inspection of histograms to ascertain whether distributions of variables approximated a normal distribution. Specific data were analysed as follows: 1) Chi-square [χ2] analysis was used to determine the relationships between categorical variables; 2) Mann-Whitney U tests were used to compare two groups where dependent variables were ordinal (e.g. usage levels between male and female participants), and independent samples t-tests were used if dependent variables were continuous and approximated a normal distribution; and 3) Spearman’s correlation analysis was used to assess the relationships between pairs of two continuous or ordinal variables - this was chosen over Pearson’s correlation because key variables were either ordinal or not normally distributed.

Survey response rates were not calculable due to the methods of recruitment, meaning it was impossible to assess the exact number of people the survey adverts had reached. However, numbers of respondents were reported.

## Results

Data from a total of 207 eligible respondents (148 female and 59 male) were analysed in this study. Typical reasons for exclusion were: non-consent to participation (3), outside geographical range (7), and incomplete responses (47). The majority (75.1%) of participants were between 18 and 34 years of age. The median BMI recorded was 23.9 (IQR 5.2, upper boundary 26.7 and lower boundary 21.5). Handedness was reported in trend with the general population, with 87.9% reporting a right-handed preference, 9.2% left-handed and 2.9% indicating they were ambidextrous (Table 1).

**Table 1:** Characteristics of participants

	n	%
Gender		
Female	148	71.5
Male	59	28.5
Total	207	100
Age (years)		
18–24	96	46.4
25–34	58	28
35–44	17	8.2
45–54	23	11.1
55–64	7	3.4
65+	4	1.9
Total	205	99
BMI		
< 18.5	6	2.9
18.5–24.9	115	55.6
25–29.9	59	28.5
30–39.9	24	11.6
Total	204	98.6
Handedness		
Right	19	9.2
Left	182	87.9
Ambidextrous	6	2.9
Total	207	100

Note: n refers to the number of participants who reported the particular characteristics

### Smartphone and tablet device use

Usage for smartphones and tablet devices was recorded in terms of frequency (times per day a device was used), duration (average duration of time spent in one session of device use) (Table 2) and purpose of use. Frequencies and durations of device use were categorised into ranges, to which a category number between 0 and 6 was assigned. Increasing category numbers reflect increased device use (Table 2). The product of the frequency category number and session duration category number was calculated to derive an overall *device usage level* (between 0 and 36) for each respondent. This reflected the total daily usage of each device type by each respondent (Table 3).

**Table 2:** Distribution of device usage - daily frequencies and average session durations for smartphones and tablet devices

Frequency category for device use	Daily frequency of device use (times per day)	Smartphone users (n)	Tablet users (n)	Session duration category	Typical duration per usage session (minutes)	Smartphone users (n)	Tablet users (n)
**0**	None reported	0	42	**0**	None reported	0	67
**1**	0–5	14	146	**1**	0–5	63	76
**2**	6–10	29	15	**2**	6–10	67	10
**3**	11–20	50	3	**3**	11–20	45	27
**4**	21–50	55	0	**4**	21–50	9	19
**5**	51–100	21	0	**5**	51–100	9	5
**6**	Too many to count	38	1	**6**	More than 2 h	14	3
	Total	207	207		Total	207	207

Note: n refers to the number of participants who reported particular items

**Table 3:** Distributions of total daily device usage levels among respondents: Smartphones and Tablet devices

Device usage level	Smartphone use (n)	Smartphone use (%)	Smartphone use: cumulative (%)	Tablet use (n)	Tablet use (%)	Tablet use: cumulative (%)
0	0	0.0	0.0	67	32.4	32.4
1	7	3.4	3.4	75	36.2	68.6
2	13	6.3	9.7	9	4.3	72.9
3	15	7.2	16.9	19	9.2	82.1
4	22	10.6	27.5	15	7.2	89.4
5	10	4.8	32.4	4	1.9	91.3
6	39	18.8	51.2	8	3.9	95.2
7	0	0	51.2	0	0	95.2
8	19	9.2	60.4	5	2.4	97.6
9	10	4.8	65.2	1	0.5	98.1
10	7	3.4	68.6	0	0	98.1
11	0	0	68.6	0	0	98.1
12	32	15.5	84.1	2	1.0	99.0
13	0	0	84.1	0	0	99.0
14	0	0	84.1	0	0	99.0
15	6	2.9	87.0	1	0.5	99.5
16	2	1.0	87.9	1	0.5	99.5
17	0	0	87.9	0	0	99.5
18	7	3.4	91.3	1	0.5	100.0
19	0	0	91.3	Total 207	100.0	100.0
20	2	1.0	92.3			
21	0	0	92.3			
22	0	0	92.3			
23	0	0	92.3			
24	6	2.9	95.2			
25	1	0.5	95.7			
26	0	0	95.7			
27	0	0	95.7			
28	0	0	95.7			
29	0	0	95.7			
30	3	1.4	97.1			
31	0	0	97.1			
32	0	0	97.1			
33	0	0	97.1			
34	0	0	97.1			
35	0	0	97.1			
36	6	2.9	100.0			
Total	207	100.0				

Note: n refers to the number of participants reporting particular levels of device usage

As seen in Table 3, 100% of participants used a smartphone and 67.6% also used a tablet device. Table 3 also shows that 3.4% of participants used a smartphone negligibly (device usage level of 1), thus 96.6% were significant smartphone users. However, a far greater proportions of participants reported no use, or negligible use, of a tablet: 32.4% reported no use and 36.2% indicated they used a tablet negligibly. Thus, 31.4% of participants were considered significant tablet users and 68.6% were considered smartphone-only users (i.e. no significant tablet use).

Nearly all participants (206 of 207) reported using their devices for leisure, 66.7% for work and 61.4% for education while 3.9% reported using their devices for ‘other’ purposes, which mainly included descriptions of leisure activities or daily living activities such as banking or obtaining weather updates.

Distributions of device usage levels for males and females were similar for smartphone and tablet device use, when assessed by visual inspection. The difference in device usage levels between males (smartphone mean rank = 92.7, tablet mean rank = 92.1) and females (smartphone mean rank = 108.5, tablet mean rank = 108.7) was not statistically significant (Mann-Whitney U test: smartphone U = 3698, z = − 1.729, *p* = .083; tablet U = 3666, z = − 1.879, *p* = .060).

### Symptom prevalence and onset time, and device usage levels

**Fig. 2:**
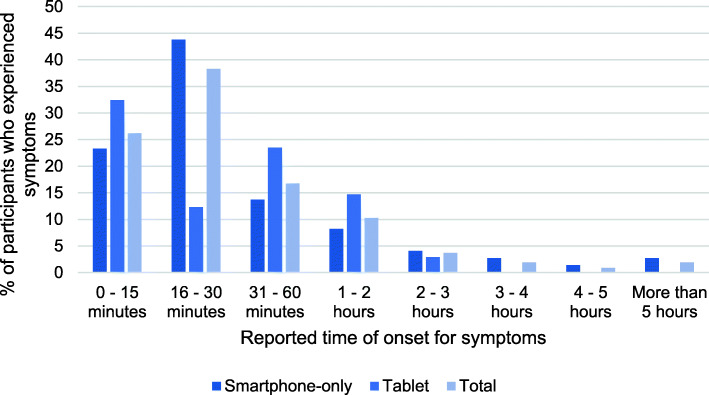
Proportions of participants with symptoms who began to experience them within each time interval

**Fig. 3:**
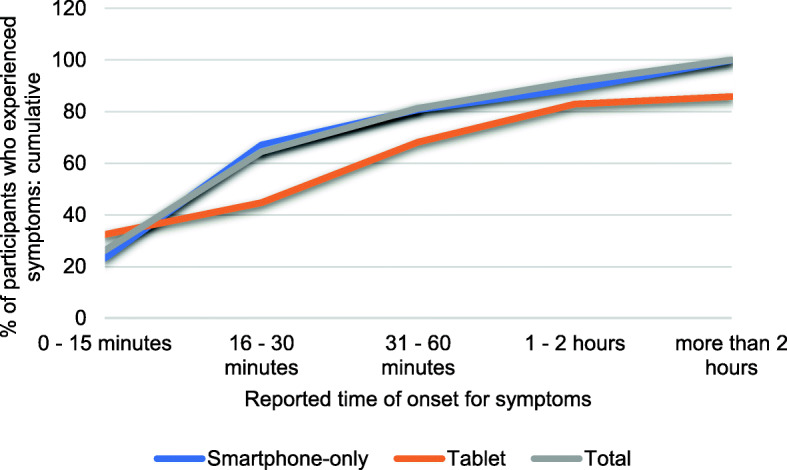
cumulative proportions of participants with symptoms who began to experience them within each time interval

Of the 207 participants, 59.9% reported musculoskeletal symptoms during or after device use. However, of these, 34.3% also reported pain or injuries from other causes but this was not analysed further in this study which should be considered in further studies. Symptoms tended to increase in frequency up to a threshold of device usage level and then plateau. Most participants who reported symptoms began to experience them either within the first 15 min of use (26.2%), or within the 15–30 min time period (38.3%). Together, 64.5% of symptomatic participants began to experience their symptoms within the first 30 min of usage. Furthermore, 73.8% of symptomatic users did not begin to experience symptoms until after 15 min of device usage (Fig. 2and Fig. 3).

### Differences in patterns of musculoskeletal symptoms between smartphone-only and tablet device users

Among smartphone-only users, 61.3% (87 of 142) experienced musculoskeletal symptoms, compared to 56.9% (27 of 65) of significant tablet device users, and this difference did not reach significance (χ2 = 0.350, *N* = 207, *p* = 0.554). However, Fig. 2 indicates more symptomatic smartphone-only users experienced their symptoms within the first 30 min of use (67.1%) than symptomatic tablet users (58.8%).

Both device-type groups reported their most prevalent type of symptom to be stiffness (29.4% of symptomatic smartphone-only users and 29.6% of symptomatic tablet users), followed by discomfort (27.6 and 26.5%), aches (16 and 22.7%), pain (14.3 and 11.3%), pins and needles (6.8 and 5.5%), and numbness (5.9 and 4.4%). Where pain was the symptom experienced, the mean (±SD) level of pain reported was 2.9 (±1.4) out of 10 on the VAS, and the reported levels of pain ranged between 1 and 6 on the 10-point VAS. The mean pain level for smartphone-only users was 3.01 (±1.5) and for tablet users was 2.77 (±1.4). The difference in mean pain levels between smartphone-only users and tablet device users was not statistically significant (t = −.819, *N* = 207, *p* = .414).

**Fig. 4:**
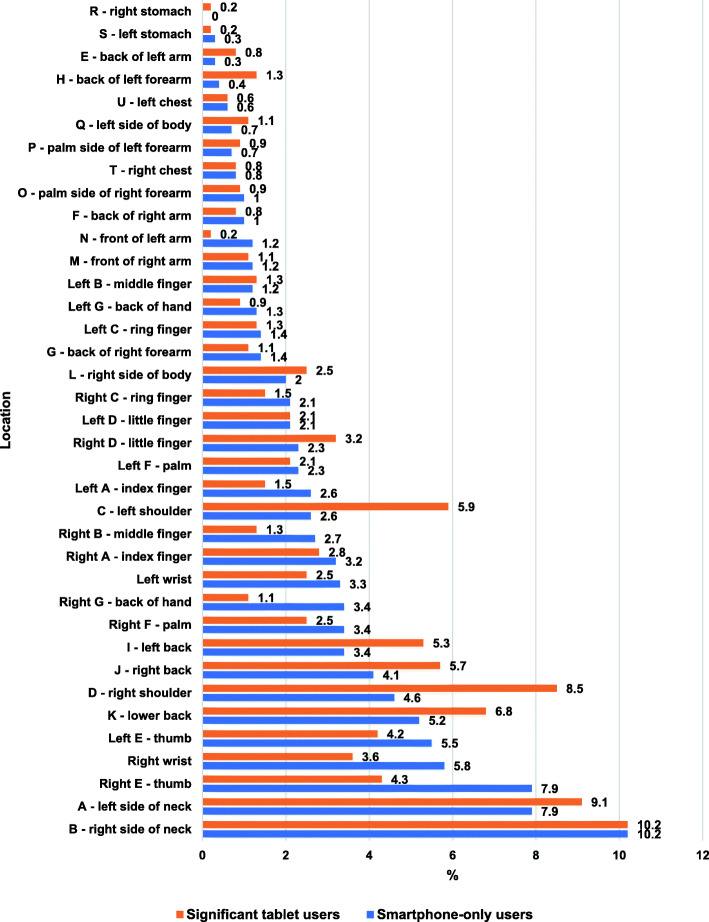
Percentages (%) of all symptoms reported by participants in the respective user group which were located at each body site. Note: the capital letter in front of each region label corresponds to the labels in the body chart in Fig. 1

Overall, symptom locations were similarly distributed in smartphone-only and tablet device users (Fig. 4). Among smartphone users who reported symptoms, 18.1% of symptoms were experienced in their neck (right = 10.2%, left = 7.9%). Similarly, 19.3% of symptoms reported by tablet device users affected their neck (right = 10.2%, left = 9.1%). However, some notable differences were observed between these two groups. Posterior aspects of both upper arms, forearms and shoulders (particularly left shoulder) were more common sites of symptoms among the tablet device users than the smartphone-only users. Conversely, the lower back, wrists (particularly right wrist), right hand and both thumbs (left thumb to a lesser extent) were more prominent sites of symptoms in smartphone-only users (Fig. 4).

### Associations of gender, age, BMI and handedness with musculoskeletal symptoms

Ninety-four (94) of 148 female participants (64%) and 30 of 59 male participants (51%) reported musculoskeletal symptoms associated with device use in the previous two-week period. This gender difference was not statistically significant (χ2 = 2.818, *N* = 207, *p* = .116). The relationships between symptom occurrence and participants’ BMI levels or ordered age categories (18–24, 25–34, 35–44, 45–54, 55–64, or 65+ years) were very weak and did not reach statistical significance in the Spearman’s correlation analyses (BMI – r_*S*_ = .101, *N* = 204, *p* = .151; Age – r_*S*_ = .038 *N* = 205, *p* = .593).

Of right-handed participants, 59.9% experienced symptoms, compared to 52.6% of left-handed participants, and notably, 83.3% (5 of 6) of ambidextrous participants experienced symptoms. Disregarding those who were ambidextrous (only 2.9% of participants), there was no significant association of right or left handedness with symptom experience (χ2 = .375, *N* = 201, *p* = .540).

Age was also investigated alongside duration of device usage by smartphone and tablet device users, as potential influences on symptom experience. Age was dichotomised to ensure minimum required observed cell counts for age categories in the Chi-square [χ2] tests of association. Usage time was also dichotomised for the same reason and a 30 min cut-point was chosen due to the significance of the 30 min time-point for symptom development based on the data in Fig. 2 and in other types of sedentary activities reported in previous studies [2, 7, 8, 16]. There was no significant association in smartphone users between symptom experience and duration of usage in either 18–24 year-olds (χ2 = .412, *N* = 109, *p* = .521) or over 25 year-olds (χ2 = .392, *N* = 96, *p* = .531). However, Table 4 shows a statistically significant association was observed between duration of tablet use and symptoms, such that tablet users with use durations > 30 min were more likely to experience symptoms than users with shorter durations of tablet use (χ2 = 4.083, *N* = 140, *p* = .043). Further analysis revealed that this association reached significance in the 18–24 year-old group (χ2 = 4.723, *N* = 63, *p* = .030) but not in the over 25 year-old age group (Table 4).

**Table 4:** Associations in tablet users between dichotomised durations of usage per session (≤ 30 min or > 30 min) and symptom experience, by age group. Note: n refers to the number of participants in the specified category

	Tablet use ≤30 mins % (n) with symptoms	Tablet use > 30 mins % (n) with symptoms	*P* value
All ages	56.6 (27)	77.8 (113)	.043*
18–24	52.2 (17)	82.4 (46)	.030*
25+	58.5 (10)	70 (65)	.488

### Duration and types of postures adopted during device usage, operational methods and musculoskeletal symptoms

In smartphone and tablet device users, the most common position of use was sitting; 27.5% of smartphone users and 38.1% of tablet users adopted this position at some time while using their device. This position also had the highest symptom prevalence in both device-type groups (55.8 and 50.8%), and equal prevalence with lying on back for smartphone users (55.8%; Table 5). Those who reported adopting a standing position at some point during their device usage had a lower prevalence of symptoms (49.5% of smartphone users and 36.4% of tablet users).

**Table 5:** Symptom prevalence and posture adopted during device usage, by device type

Adopted posture	Smartphone user symptom prevalence (of n)	Tablet user symptom prevalence (of n)
Sitting	55.8% (of 165)	50.8% (of 61)
Standing	49.5% (of 109)	36.4% (of 11)
Lying on back	55.8% (of 95)	47.4% (of 19)
Lying on side	55.3% (of 85)	47.4% (of 19)
Lying on front	50% (of 14)	50% (of 38)
Varying positions	52.5% (of 99)	50% (of 12)

Note: n refers to the number of participants who adopted this particular position some of the time when using the specified device

Table 6 showed how frequency of changes in position related to symptom prevalence. The lowest prevalence of symptoms (47.7% in smartphone users and 37.5% in tablet users) occurred in people who changed positions every 5 min, disregarding the 5 tablet users who reported never changing position. The highest prevalence of symptoms occurred in those who typically changed positions every 30 min for smartphone users (81.8%) and every 30–60 min (73.3%) for tablet users. Increased times between position changes tended to be associated with increased percentages of people experiencing symptoms, up until changing position every 30 min for smartphone users and 30–60 min for tablet users. After this point, symptom prevalence appeared to reduce; however, this may be an artefact of the small numbers of participants in those longer time ranges (Table 6).

**Table 6:** Frequency of changes in position and symptom prevalence, by device type

Frequency of change in position	Smartphone user symptom prevalence (of n)	Tablet user symptom prevalence (of n)
Every 5 mins	47.7% (of 65)	37.5% (of 8)
Every 10 mins	51.9% (of 53)	41.2% (of 17)
Every 15 mins	63.2% (of 38)	50% (of 18)
Every 20 mins	64% (of 25)	60% (of 10)
Every 30 mins	81.8% (of 11)	50% (of 10)
Every 30–60 min	71.4% (of 7)	73.3% (of 15)
Hourly or longer	50% (of 6)	50% (of 2)
Don’t change	54.5% (of 11)	20% (of 5)

Note: n refers to the number of participants who changed position with the specified timeframe when using the specified device

Those who operated a device while not holding the device at all constituted the group that was least likely to experience symptoms (44.4% of smartphone users and 43.3% of tablet users; Table 7). Left hand only holders appeared to be most at risk of experiencing symptoms (83.3 and 100%).

**Table 7:** Symptom prevalence by device type and method of holding device

Holding device	Smartphone user symptom prevalence (of n)	Tablet user symptom prevalence (of n)
Right hand only	61.4% (of 44)	50% (of 4)
Right hand mainly	56.8% (of 81)	60% (of 5)
Both hands equally	57.9% (of 38)	52.9% (of 34)
Left hand mainly	48.1% (of 27)	50% (of 10)
Left hand only	83.3% (of 6)	100% (of 3)
Don’t hold it – use a cradle, stand or other	44.4% (of 9)	43.3% (of 30)

Note: n refers to the number of participants who held the specified device in the specified manner

Device operation using both hands equally tended to be lower risk for symptoms among smartphone users (46.2% prevalence of symptoms) than other operational methods (Table 8). This operational method was also lower risk for tablet device users (38.9% prevalence), but right hand only operation was the lowest risk method of operation among tablet device users (38.5% prevalence).

**Table 8:** Symptom prevalence by device type and method of device operation

Method of operation of device	Smartphone user symptom prevalence (of n)	Tablet user symptom prevalence (of n)
Right thumb only	58.3% (of 72)	50% (of 2)
Right hand only	60.7% (of 28)	38.5% (of 13)
Right hand mainly	56.8% (of 81)	52.9% (of 34)
Both hands equally	46.2% (of 13)	38.9% (of 18)
Both thumbs equally	53.8% (of 26)	100% (of 5)
Left hand mainly	100% (of 2)	(0)
Left thumb only	100% (of 4)	(0)

Note: n refers to the number of participants adopted this particular method of device operation

## Discussion

This study investigated the prevalence and patterns of musculoskeletal symptoms among smartphone and tablet device users using an online questionnaire. It also explored patterns in the usage of smartphone and tablet devices in terms of time, postures, operational methods and purposes. It was found that the majority of mobile device users experienced symptoms during device use. Most symptoms began within the first 30 min of device usage, and particularly between 15 and 30 min of usage. There was a significant positive association between tablet (but not smartphone) usage duration and symptom prevalence. No significant differences between smartphone-only and tablet users in usage and symptom experience were found. Symptoms occurred least frequently in device users who sometimes adopted a standing posture, changed position frequently, did not hold their device when using it, or used both hands equally to operate their device.

### Musculoskeletal symptoms: onset and duration of device use

A majority of participants experienced symptoms over the previous two-week usage period. This finding is consistent with a previous systematic review of the symptoms associated with handheld device use conducted by Xie et al. [22]. This study showed that symptoms most frequently began between 15 and 30 min from commencing use of a smartphone or tablet device. This finding suggests that if mobile device users were to limit their usage to 15 min in any one session, more than 70% would avoid the onset of symptoms. There are no previous studies with which to directly compare these results regarding the threshold time at which symptoms begin during device use. However, the findings are comparable with recommended timeframes for sedentary activities, in order to reduce risks of adverse health impacts. For example, accumulating a sedentary time in each session of less than 29 min was associated with a reduced risk for all-cause mortality according to Diaz et al. [7]. These findings also support the current Australian recommendations for minimising sedentary activity [4]. However, there is no specific timeframe given in those recommendations.

Previous literature has reported a lack of clear association between device usage time and symptom prevalence [22]. Our findings for smartphone users, indicating no significant difference in symptom prevalence between those who used their smartphone for more than 30 min and those who used it for 30 min or less, are consistent with the findings of Xie et al. [22]. Conversely, our findings for tablet users differed - a significant association was observed between symptom prevalence and dichotomised usage times, such that tablet users who typically used their device for > 30 min per session more often experienced symptoms than those who used their tablet for < 30 min. This relationship between session duration and symptom prevalence in tablet users was particularly evident in the younger, 18–24 year-old, group, and this apparent age-dependence of the relationship between session duration and symptom prevalence warrants further research in larger samples of tablet users, including children who were not part of this study.

### Differences between smartphone and tablet device users

As expected from the study inclusion criteria, all participants used a mobile device of some sort; all used a smartphone (96.6% significantly) but only a third of participants also used a tablet device significantly. Smartphone users and tablet users experienced similar rates of musculoskeletal symptoms.

Smartphone-only users and significant tablet device users both reported their most prevalent symptom to be stiffness, followed by discomfort, aches, pain, pins and needles, and numbness. The most common location of symptoms for both device-types was the neck, with almost one fifth of all symptoms reported occurring in this region. This is consistent with observations from a high-quality systematic review, which ascertained that neck complaints were the most prevalent symptoms, occurring in 17.3 to 67.8% of mobile device users [22]. In the current study, right sided neck symptoms were more common than left sided symptoms in smartphone users, particularly. To the best of our knowledge, no previous investigation has distinguished between right and left symptoms of the neck. It is possible that the ‘right side of neck’ was more often symptomatic due to most smartphone users also holding their device in their right hand and thus increasing the muscle strain on that side. In contrast, tablet device users more commonly reported holding the device in both hands equally and this could explain the more similar distribution of symptoms observed across right and left sides of the neck in the tablet device group. Future laboratory-based studies should be carried out to further investigate effects of these factors on symptoms.

Symptoms in the significant tablet device user group tended to localise more proximally than in the smartphone-only user group; arms (bilateral backs of upper arms and forearms) and shoulders (particularly left shoulder) were more prominent symptom locations among the tablet device users than the smartphone-only users. To the best of our knowledge, this is the first study to distinguish tablet devices from smartphone devices in a study investigating symptom location and type.

### Age, BMI, gender, handedness and symptom prevalence

Aside from the age-specific relationship between duration of tablet use and symptom prevalence discussed in section 4.1, age was not significantly associated with symptom experience.

There were also no statistically significant associations between gender, BMI, or handedness and symptom prevalence. The finding regarding gender differences contrasts with findings of previous research, which found female tablet users were 2.1 times more likely to have symptoms than males [13]. This may require further investigation.

### Posture, operational methods and symptom prevalence

Symptom prevalence tended not to change much based on position when using devices. However, symptoms occurred least frequently in device users who sometimes adopted a standing posture. Sitting had the highest symptom prevalence in both device-type groups (and was the most commonly adopted posture) and an equivalent prevalence of symptoms was recorded for smartphone users lying on their back. Previous literature reports posture during device use with neck flexion as a prominent risk factor for musculoskeletal symptoms [1, 6, 9]. Vasavada et al. [21] reported that neck flexion during device usage increased the demand on neck muscles by up to 3–5 times when compared with a neutral position. It may be beneficial for further studies to take observational measures for postures and self-reported symptoms.

The lowest prevalence of musculoskeletal symptoms occurred in people who changed positions more frequently (every 5 min). The most common timeframe for changing positions was every 30 min for smartphone users and every 30–60 min for tablet device users. Increased time between changing positions tended to increase percentage of people experiencing symptoms up until every 30–60 min for smartphone users and hourly or longer for tablet users. After this point, symptom prevalence reduced and plateaued; this may be due to the small number of participants in those longer time ranges.

Participants who did not hold the device at all (i.e. used a cradle or other external support for the device) were least likely to experience symptoms. Conversely, left hand only holders were most at risk of experiencing symptoms, however, this may have been an artefact of small cell numbers. Operating the device using both hands equally tended to be lower risk for symptoms among smartphone users than other operational methods. This operational method was also low risk for tablet device users, but right hand only was the lowest risk method for tablet device users. This may be due to single-handed usage increasing asymmetry of muscular demands and strain.

### Strengths and limitations

The research design was carefully considered to ensure the validity of findings. Particular design issues considered included: i) differentiating between smartphones and tablet devices; ii) ensuring a broad population was surveyed (age ranges 18 to over 65); iii) gathering and considering in analyses the characteristics of participants; iv) including a body chart for reporting of symptoms; and (v) ensuring the recall period was short (two-weeks) to minimise recall bias in findings. To the best of the authors’ knowledge, this research is also the first of its type conducted in the eastern states of Australia.

The study also has some limitations. It only measured musculoskeletal symptoms and not any other impact of device use, including those affecting a person’s psychological health. Information regarding the specific activities that were being undertaken on devices was not assessed. No intervention occurred in this research project - the study was limited to analysing retrospective self-reported information obtained through the online questionnaire only. This survey did not include some body locations such as the eye, which have been found to be a location of strain and discomfort in previous literature [18]. The sample included only adults from Australia and findings should not be assumed to be generalizable to children or to adults from other regions of the world, where conditions may differ.

The subjective nature of self-reported symptoms may result in self-report bias arising for example from perceptions of social desirability or specific responses, the two-week recall period or selective recall. The recall bias associated with this study may have been an issue for some participants who have irregular use patterns. Participants were able to assess their usage using screen time apps which was encouraged, however not enforced. Future studies may benefit from enforcing the use of a screen time app for reporting, a logbook or a laboratory-based study with more objective measures taken and external assessors of device usage.

The anonymous nature of the survey may have reduced the impact of social desirability bias on participant responses. The survey was also only available online, which limited access for those without internet accessibility. Although the tools employed to gather data on pain location and intensity were relatively simple and intuitive, it is possible that lack of familiarity of respondents with these tools may have affected the data.

## Conclusion

The findings of this study can contribute to the formulation of evidence-based guidelines to reduce experiences of musculoskeletal symptoms among smartphone and tablet device users and guide future research into the possible risks associated with smartphone and tablet device use. Specifically, the significance of duration of tablet use and younger age on experience of musculoskeletal symptoms has been identified, along with a range of posture and device-operation factors that affect experiences of musculoskeletal symptoms. Symptom onset most commonly occurred between 15 and 30 min from commencement of device use, in both device-type groups. If participants were to have limited their usage to 15 min per session, over 70% of participants would have avoided symptoms.

Based on the findings, it is reasonable to recommend that adult mobile device users cap their usage at less than 15 min per session whenever possible, avoid sustained static postures during device use, use external supports for their device, and use both hands equally to operate the device, in order to minimise their risk of experiencing musculoskeletal symptoms.

These findings can also be used to support future studies on the factors impacting musculoskeletal symptoms and further advance knowledge of the ever-evolving issues surrounding mobile device use and human health. Further research is warranted to evaluate the effectiveness of these recommendations and to investigate their relevance for children.

## Supplementary Information

**Additional file 1.**

## Data Availability

The datasets generated and analysed during the current study available from the corresponding author on reasonable request.
