# Supplementary material for: Musculoskeletal symptoms among adult smartphone and tablet device users: a retrospective study
Source: Arch Physiother. 2021 Jan 9;11:1. doi: 10.1186/s40945-020-00096-6 (PMC7796547; doi:10.1186/s40945-020-00096-6)
Supplement: Supplementary file 1 — Additional file 1. [file 40945_2020_96_MOESM1_ESM.pdf]

Physical Impacts of Mobile Device Usage

Participant Information

Invitation

You are invited to participate in a research project titled: "Musculoskeletal symptoms among smartphone and tablet device users".

This study is about smartphone and tablet device usage and associated patterns of musculoskeletal symptoms (physical symptoms in your muscles, bones, or joints) among adults. The study is being conducted by Ms Ella Thorburn, Physiotherapy Honours student at Charles Sturt University; Dr Shaun Wang, PhD (Biomedicine), Charles Sturt University; Prof Rodney Pope, Physiotherapist, Charles Sturt University.

Before you decide whether or not you wish to participate in this study, it is important for you to understand why the research is being done and what it will involve. Please take the time to read the following information carefully and discuss it with others if you wish.

What is the purpose of this study?
This study is about smartphone and tablet device usage, including duration, and patterns of musculoskeletal symptoms such as pain, discomfort, aching, stiffness, pins and needles or numbness. We will also compare the patterns of musculoskeletal symptoms between smartphone and tablet device users.

Why have I been invited to participate in this study?
We are seeking any adult (over the age of 18) who uses a smartphone or tablet device and is able to access this online survey about device use and possible experiences of musculoskeletal symptoms. If you are not over 18 years old or do not use a smartphone or tablet device, then please do not respond.

What does this study involve?
If you agree to participate, you will be asked to answer questions which will take around 15 minutes to complete. Further, you will be asked to access this survey on three different days over a period of two weeks. You may choose which days you would like to access the survey, but please complete the questions at the end of those days.

On the first day you will complete Part 1 which will include questions on general smartphone and tablet device use and some descriptive information about you (such as your age and height etc.). You will also be asked to create your own unique identification code to allow us to connect your Day 1, 2 and 3 responses to each other. This will not identify you and no one else will know your code.

For Part 2, which is to be completed on the second and third days, you will need to log your use of mobile devices on that day only. We need you to record each time you use your smartphone and/or tablet device and the duration of usage each time, along with any musculoskeletal symptoms that you feel. It would be best to view the questions prior to recording this information and completing the

specific daily usage section (Part 2) so that you are prepared.

Are there risks and benefits to me in taking part in this study?
There will be no direct benefit to you in participating in this research. Your decision to participate or not will have no direct consequence for you. However, any findings regarding device usage and musculoskeletal symptoms may contribute to guides for safe use and prevention of symptoms which participants may be experiencing.

There are no foreseeable risks involved in completing this survey. The survey is anonymous, and all your responses will be kept confidential. No personally identifiable information will be associated with your responses or included in any reports of these data. It is recommended that if this survey raises any concerns regarding physical health, you seek appropriate advice from your GP or a physiotherapist.

How is this study being paid for?
This study is an Honours project which does not require funding. It is run through the Faculty of Science, Charles Sturt University (CSU).

Will taking part in this study (or travelling to) cost me anything, and will I be paid?
There is no cost to complete the survey. No reimbursement or payments to participants will be provided.

What if I don't want to take part in this study?
Participation in this research is entirely your choice. Only those people who give their informed consent will be included in the project. Whether or not you decide to participate is your decision and will not disadvantage you.

What if I participate and want to withdraw later?
You can stop completing the survey at any time. However, data you have entered cannot be withdrawn because it is anonymous.

How will my confidentiality be protected?
All information collected in this survey is protected by SurveyMonkey'sPrivacy Policy and Security Statement. Downloaded data will be stored securely on password-protected computers belonging to CSU researchers. The survey is anonymous, and it will not be possible to identify you from your answers. Your non-identified, anonymous responses will be stored securely in an electronic spreadsheet for a period of at least five years.

What will happen to the information that I give you?
The data from this study will be reported in a non-identifying form in a dissertation that will be provided to CSU academics for Ms Ella Thorburn's degree. The researchers also hope to present the findings of this study in scholarly journals and at academic conferences. Multiple reports and resultant publications are expected.

If you have a particular question about the findings of our study, you are invited to put this question in an email to Ms Ella Thorburn at ella.thorburn.95@gmail.com and a relevant summary will be sent to you. Individual participants will not be identified, and all data will be reported in an aggregate manner.

What should I do if I want to discuss this study further before I decide?
Please contact Ms Ella Thorburn at ella.thorburn.95@gmail.com or on 0434604107 from whom potential

participants can obtain further information about the project.

Who should I contact if I have concerns about the conduct of this study?

CSU's Human Research Ethics Committee has approved this project (protocol number H18271). If you have any complaints or reservations about the ethical conduct of this project, you may contact the Committee through the Executive Officer:

The Executive Officer
Human Research Ethics Committee
Tel: (02) 6338 4628
Email: ethics@csu.edu.au

Any issues you raise will be treated in confidence and investigated fully and you will be informed of the outcome.

Thank you for considering this invitation.

Please print this information if you wish to keep a copy for your records.

*** 1. Do you wish to participate as outlined above and give your informed consent?**

- ◯ Yes
- ◯ No

Charles Sturt University

Physical Impacts of Mobile Device Usage

First, second or third entry

* **2. Are you completing this survey for a second or third time?**

- No, this is the first time
- Yes, second time
- Yes, third time

Charles Sturt University

Physical Impacts of Mobile Device Usage

Part 1: Demographics

Please create a code that will allow us to connect your responses.

The code should be unique to you and easy for you to remember so you can enter the same code on Days 2 and 3.

As a suggestion, you could use your initials and the last three digits of your phone number.

*** 3. Please create and enter your unique identification code:**

4. What is your gender?

- Female
- Male
- Non-binary
- Other

5. In what age group are you?

6. In what country do you live?

Charles Sturt University

Physical Impacts of Mobile Device Usage

Australia - postcode

7. What is your postcode?

Charles Sturt University

Physical Impacts of Mobile Device Usage

Part 1: BMI and handedness

8. What is your height (in cms)?

9. What is your weight (in kgs)?

10. Which is your dominant hand?

- Left hand
- Right hand
- Both equal (ambidextrous)

Charles Sturt University

Physical Impacts of Mobile Device Usage

Part 1: Device Usage

11. On average, how many times a day would you use a smartphone and/or tablet device?

	Times
Smartphone	
Tablet device	

12. On average, for how long would you use the smartphone and/or tablet device on each of these occasions?

	Length of Use (Minutes)
Smartphone	
Tablet device	

13. Do you use your smartphone and/or tablet device for work, education or leisure? (Please indicate all that apply)

- [ ] Work
- [ ] Education
- [ ] Leisure
- [ ] Other (please specify)

Physical Impacts of Mobile Device Usage

Part 1: Muscular or skeletal symptoms in past 2 weeks

14. Have you experienced any of the following physical symptoms in any of your muscles, bones or joints during or after smartphone or tablet device usage in the *past 2 weeks*?

- **Pain**
- **Discomfort**
- **Stiffness**
- **Aches**
- **Pins and needles**
- **Numbness**

○ Yes

○ No

Physical Impacts of Mobile Device Usage

Part 1: Hand and wrist symptoms in past 2 weeks

Hands and wrists

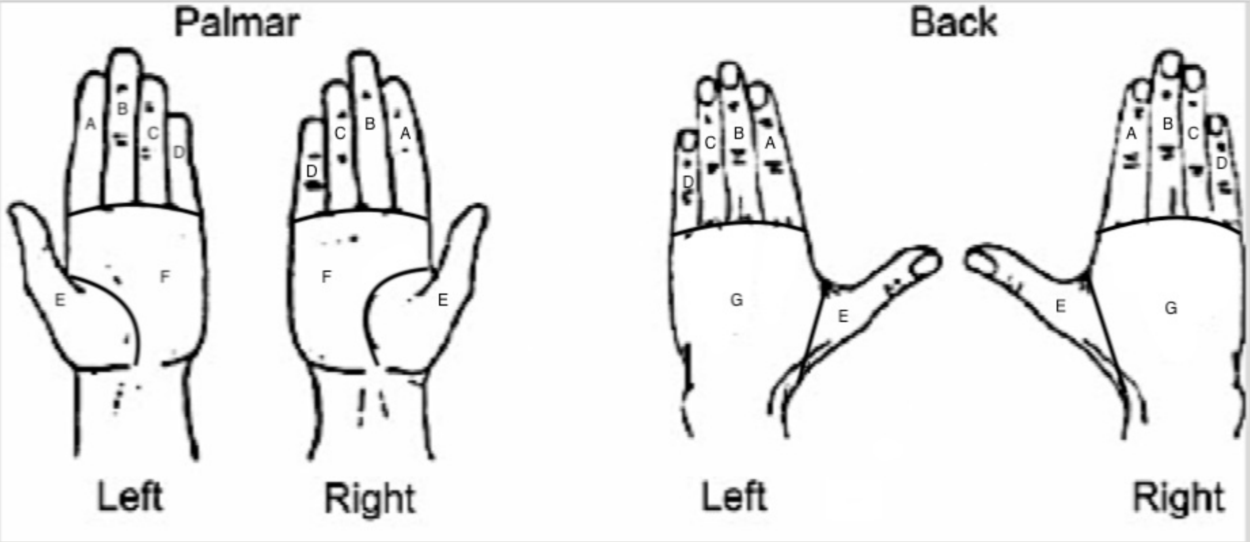

15. Using the letters on the above diagram, please indicate where and what type/s of symptoms have been felt *in the past two weeks*:
(More than one response per row can be selected if applicable)

	Pain	Discomfort	Stiffness	Aches	Pins and needles	Numbness
Left A - index finger	☐	☐	☐	☐	☐	☐
Left B - middle finger	☐	☐	☐	☐	☐	☐
Left C - ring finger	☐	☐	☐	☐	☐	☐
Left D - little finger	☐	☐	☐	☐	☐	☐
Left E - thumb	☐	☐	☐	☐	☐	☐
Left F - palm	☐	☐	☐	☐	☐	☐
Left G - back of hand	☐	☐	☐	☐	☐	☐
Left wrist	☐	☐	☐	☐	☐	☐
Right A - index finger	☐	☐	☐	☐	☐	☐
Right B - middle finger	☐	☐	☐	☐	☐	☐
Right C - ring finger	☐	☐	☐	☐	☐	☐
Right D - little finger	☐	☐	☐	☐	☐	☐
Right E - thumb	☐	☐	☐	☐	☐	☐
Right F - palm	☐	☐	☐	☐	☐	☐
Right G - back of hand	☐	☐	☐	☐	☐	☐
Right wrist	☐	☐	☐	☐	☐	☐

16. Please add any further information about the symptoms you have felt in your hands or wrists in the past two weeks:

Physical Impacts of Mobile Device Usage

Part 1: Body symptoms in past 2 weeks

Body symptoms

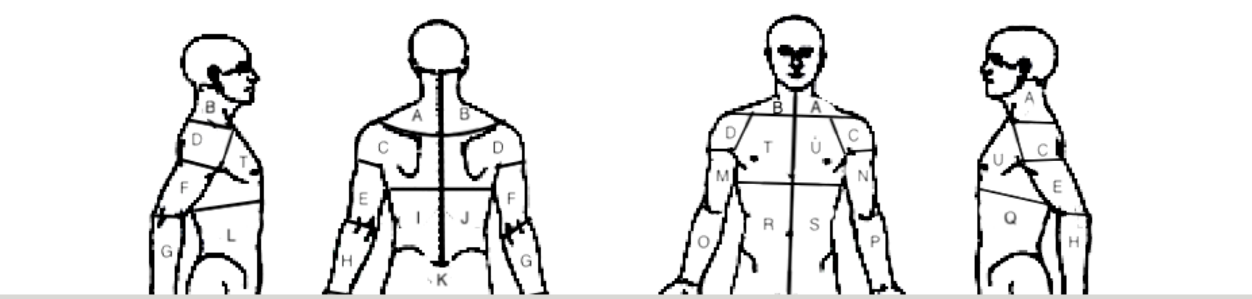

17. Using the letters on the above diagram, please indicate where and what type/s of symptoms have been felt *in the past two weeks*:
(More than one response per row may be selected if applicable)

	Pain	Discomfort	Stiffness	Aches	Pins and needles	Numbness
A - left side of neck	☐	☐	☐	☐	☐	☐
B - right side of neck	☐	☐	☐	☐	☐	☐
C - left shoulder	☐	☐	☐	☐	☐	☐
D - right shoulder	☐	☐	☐	☐	☐	☐
E - back of left arm	☐	☐	☐	☐	☐	☐
F - back of right arm	☐	☐	☐	☐	☐	☐
G - back of right forearm	☐	☐	☐	☐	☐	☐
H - back of left forearm	☐	☐	☐	☐	☐	☐
I - left back	☐	☐	☐	☐	☐	☐
J - right back	☐	☐	☐	☐	☐	☐
K - lower back	☐	☐	☐	☐	☐	☐
L - right side of body	☐	☐	☐	☐	☐	☐
M - front of right arm	☐	☐	☐	☐	☐	☐
N - front of left arm	☐	☐	☐	☐	☐	☐
O - palm side of right forearm	☐	☐	☐	☐	☐	☐
P - palm side of left forearm	☐	☐	☐	☐	☐	☐
Q - left side of body	☐	☐	☐	☐	☐	☐
R - right stomach	☐	☐	☐	☐	☐	☐
S - left stomach	☐	☐	☐	☐	☐	☐
T - right chest	☐	☐	☐	☐	☐	☐
U - left chest	☐	☐	☐	☐	☐	☐

18. Please add any further information about body symptoms you have felt in the past two weeks:

Charles Sturt University

Physical Impacts of Mobile Device Usage

Part 1: Symptoms in past 2 weeks

19. How long after starting to use your smartphone or tablet device did you experience these symptoms?

- ◯ 0 - 15 minutes
- ◯ 15 - 30 minutes
- ◯ 30 - 60 minutes
- ◯ 1 - 2 hours
- ◯ 2 - 3 hours
- ◯ 3 - 4 hours
- ◯ 4 - 5 hours
- ◯ More than 5 hours

20. Do you have any injuries or pain from any *other* causes in these areas?

- ◯ Yes
- ◯ No

21. How would you rate the worst pain you have experienced, during or after using the smartphone or tablet device in the *past 2 weeks*?

No pain | Moderate pain | Unbearable pain

Charles Sturt University

Physical Impacts of Mobile Device Usage

Part 1: Usage positions

22. What position/s are you mainly in when using your smartphone or tablet device?

	Smartphone	Tablet device
Sitting	☐	☐
Standing	☐	☐
Lying on back	☐	☐
Lying on side	☐	☐
Lying on front	☐	☐
Moving around/varying position	☐	☐

Other (please specify)

____

23. Typically, about how often do you change your position during one session using each type of device?

	Smartphone	Tablet device
Every 5 minutes	☐	☐
Every 10 minutes	☐	☐
Every 15 minutes	☐	☐
Every 20 minutes	☐	☐
Every 30 minutes	☐	☐
Between 30 - 60 minutes	☐	☐
Hourly or longer	☐	☐
Don't change	☐	☐

Charles Sturt University

Physical Impacts of Mobile Device Usage

Part 1: Holding and operating

24. How do you mainly hold the devices when using them?

	Smartphone	Tablet device
Right hand only	☐	☐
Right hand mainly	☐	☐
Both hands equally	☐	☐
Left hand mainly	☐	☐
Left hand only	☐	☐
Don't hold it - use cradle, stand, table or other rest	☐	☐

25. Which hand/s do you mainly use to operate the devices?

	Smartphone	Tablet device
Right thumb only	☐	☐
Right hand only	☐	☐
Right hand mainly	☐	☐
Both hands equally	☐	☐
Both thumbs equally	☐	☐
Left hand mainly	☐	☐
Left hand only	☐	☐
Left thumb only	☐	☐

Charles Sturt University

Physical Impacts of Mobile Device Usage

Part 1: Complete

Thank you for completing Part 1 of the survey, we look forward to having you complete Part 2 twice within the next two weeks.
(Note: Please complete the questions at the end of the day)

You may wish to view the questions in Part 2 so you are prepared but PLEASE DO NOT COMPLETE these questions now. (You will just need to enter your unique identification code)

It may be helpful to use an app to track your daily usage (e.g. RealizD - Screen Time Tracker).
On an iPhone you could use the 'screen time' function which can be found in Settings.

Charles Sturt University

Physical Impacts of Mobile Device Usage

Part 2: Day 2 or Day 3 (second or third entry of data)

Your unique identification code.

We suggested you could use your initials and the last three digits of your phone number.

* **26. What is your unique identification code:**
(You created this on Day 1)

Part 2: Specific usage - Smartphone

27. Did you use your smartphone today?

- ○ Yes
- ○ No

Charles Sturt University

Physical Impacts of Mobile Device Usage

Part 2: Specific usage - Smartphone

28. Approximately what size is your smartphone?

- ◯ 6 x 14cm (e.g. iPhone 6/6s/7/8)
- ◯ 7 x 14cm (e.g. iPhone X/Xs / Google Pixel 3)
- ◯ 7 x 16cm (e.g. iPhone XS max/7 or 8 plus/ Galaxy note 9)

29. Approximately how long *<u>in total</u>* did you spend on your smartphone *<u>today</u>*?

- ◯ Less than 15 minutes
- ◯ 15 - 30 minutes
- ◯ 30 - 60 minutes
- ◯ 1 - 2 hours
- ◯ 2 - 3 hours
- ◯ 3 - 4 hours
- ◯ 4 - 5 hours
- ◯ More than 5 hours

30. Approximately how long did you spend on your smartphone *<u>each time</u>* you used it *<u>today</u>* (in each individual session)?

- ◯ Less than 15 minutes
- ◯ 15 - 30 minutes
- ◯ 30 - 45 minutes
- ◯ 45 - 60 minutes
- ◯ 1 - 2 hours
- ◯ 2 - 3 hours
- ◯ 3 - 4 hours
- ◯ More than 4 hours

31. Is today an average day for your smartphone usage?

- ○ Yes, about average
- ○ No, I used it less than average
- ○ No, I used it more than average

Charles Sturt University

Physical Impacts of Mobile Device Usage

Part 2: Symptoms - Smartphone

32. Have you had any physical symptoms in your muscles, bones or joints during or after *smartphone* use *today*?

- ◯ Yes
- ◯ No

Physical Impacts of Mobile Device Usage

Part 2: Hand and wrist symptoms today - Smartphone

Hands and wrists

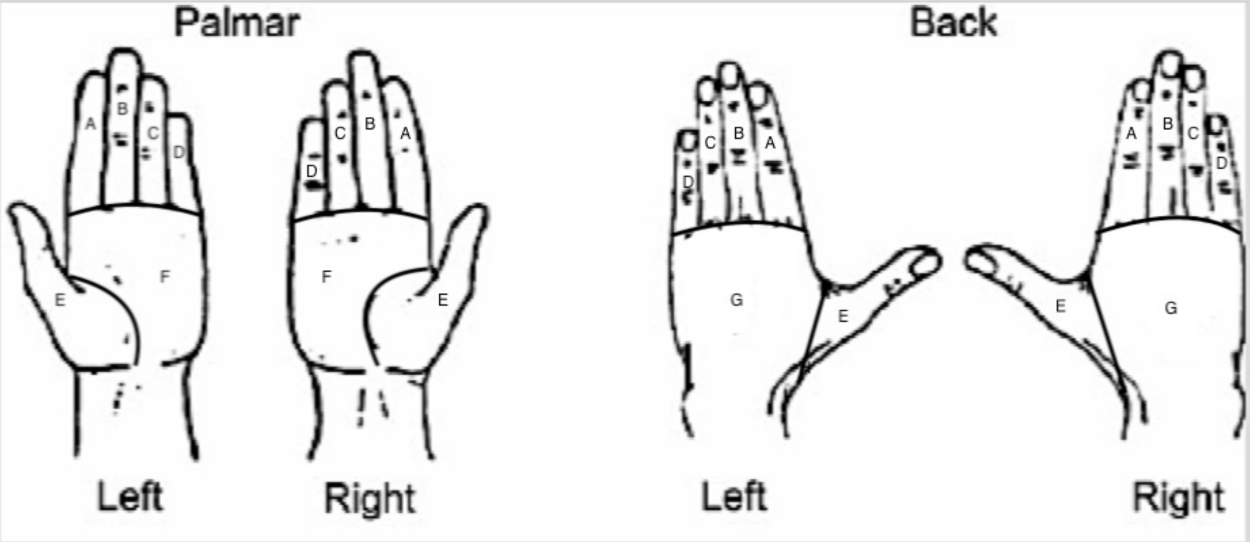

33. Using the letters on the above diagram, please indicate where and what type/s of symptoms have been felt *today*:
(More than one response per row can be selected if applicable)

	Pain	Discomfort	Stiffness	Aches	Pins and needles	Numbness
Left A - index finger	☐	☐	☐	☐	☐	☐
Left B - middle finger	☐	☐	☐	☐	☐	☐
Left C - ring finger	☐	☐	☐	☐	☐	☐
Left D - little finger	☐	☐	☐	☐	☐	☐
Left E - thumb	☐	☐	☐	☐	☐	☐
Left F - palm	☐	☐	☐	☐	☐	☐
Left G - back of hand	☐	☐	☐	☐	☐	☐
Left wrist	☐	☐	☐	☐	☐	☐
Right A - index finger	☐	☐	☐	☐	☐	☐
Right B - middle finger	☐	☐	☐	☐	☐	☐
Right C - ring finger	☐	☐	☐	☐	☐	☐
Right D - little finger	☐	☐	☐	☐	☐	☐
Right E - thumb	☐	☐	☐	☐	☐	☐
Right F - palm	☐	☐	☐	☐	☐	☐
Right G - back of hand	☐	☐	☐	☐	☐	☐
Right wrist	☐	☐	☐	☐	☐	☐

34. At the time of experiencing the above-mentioned symptoms, were you using the smartphone with one hand or two?

◯ One hand only

◯ Two hands

◯ Alternating one hand at a time

Charles Sturt University

Physical Impacts of Mobile Device Usage

Part 2: Body symptoms today - Smartphone

Body symptoms

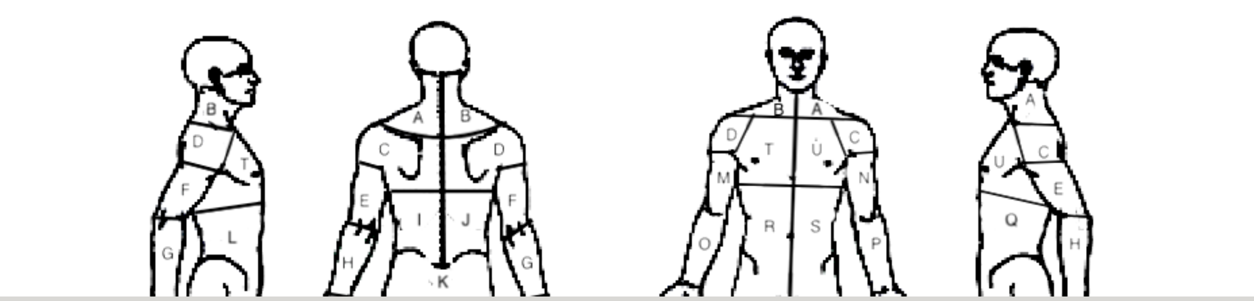

35. Using the letters on the above diagram, please indicate where and what type/s of symptoms have been felt *today*:
(More than one response per row may be selected if applicable)

	Pain	Discomfort	Stiffness	Aches	Pins and needles	Numbness
A - left side of neck	☐	☐	☐	☐	☐	☐
B - right side of neck	☐	☐	☐	☐	☐	☐
C - left shoulder	☐	☐	☐	☐	☐	☐
D - right shoulder	☐	☐	☐	☐	☐	☐
E - back of left arm	☐	☐	☐	☐	☐	☐
F - back of right arm	☐	☐	☐	☐	☐	☐
G - back of right forearm	☐	☐	☐	☐	☐	☐
H - back of left forearm	☐	☐	☐	☐	☐	☐
I - left back	☐	☐	☐	☐	☐	☐
J - right back	☐	☐	☐	☐	☐	☐
K - lower back	☐	☐	☐	☐	☐	☐
L - right side of body	☐	☐	☐	☐	☐	☐
M - front of right arm	☐	☐	☐	☐	☐	☐
N - front of left arm	☐	☐	☐	☐	☐	☐
O - palm side of right forearm	☐	☐	☐	☐	☐	☐
P - palm side of left forearm	☐	☐	☐	☐	☐	☐
Q - left side of body	☐	☐	☐	☐	☐	☐
R - right stomach	☐	☐	☐	☐	☐	☐
S - left stomach	☐	☐	☐	☐	☐	☐
T - right chest	☐	☐	☐	☐	☐	☐
U - left chest	☐	☐	☐	☐	☐	☐

Physical Impacts of Mobile Device Usage

Part 2: Symptoms and usage of smartphone

36. Please add any further information about symptoms you have felt today:

37. What position/s were you mainly in while using your smartphone?

- ☐ Sitting
- ☐ Standing
- ☐ Lying on back
- ☐ Lying on side
- ☐ Lying on front
- ☐ Moving around/varying position
- ☐ Other (please specify)

38. How long after starting to use your smartphone did you experience these symptoms?

- ◯ 0 - 15 minutes
- ◯ 15 - 30 minutes
- ◯ 30 - 60 minutes
- ◯ 1 - 2 hours
- ◯ 2 - 3 hours
- ◯ 3 - 4 hours
- ◯ 4 - 5 hours
- ◯ More than 5 hours

39. What position were you in when you experienced the symptoms?

40. How would you rate the pain you experienced, during or after using your smartphone *today*?

No pain | Moderate pain | Unbearable pain

Part 2: Specific usage - Tablet device

41. Did you use your tablet device today?

- ○ Yes
- ○ No

Part 2: Specific usage - Tablet device

42. When using your tablet device did you use an external keyboard?

- Yes, all the time
- Yes, some of the time
- No

43. Approximately how long *in total* did you spend on your tablet device *today*?

- Less than 15 minutes
- 15 - 30 minutes
- 30 - 60 minutes
- 1 - 2 hours
- 2 - 3 hours
- 3 - 4 hours
- 4 - 5 hours
- More than 5 hours

44. Approximately how long did you spend on your tablet device *each time* you used it *today* (in each individual session)?

- Less than 15 minutes
- 15 - 30 minutes
- 30 - 45 minutes
- 45 - 60 minutes
- 1 - 2 hours
- 2 - 3 hours
- 3 - 4 hours
- More than 4 hours

45. Is today an average day for your tablet device usage?

- ◯ Yes, about average
- ◯ No, I used it less than average
- ◯ No, I used it more than average

Charles Sturt University

Physical Impacts of Mobile Device Usage

Part 2: Symptoms - Tablet device

46. Have you had any physical symptoms in your muscles, bones or joints during or after *tablet device* use *today*?

- Yes
- No

Charles Sturt University

Physical Impacts of Mobile Device Usage

Part 2: Hand and wrist symptoms today - Tablet device

Hands and wrists

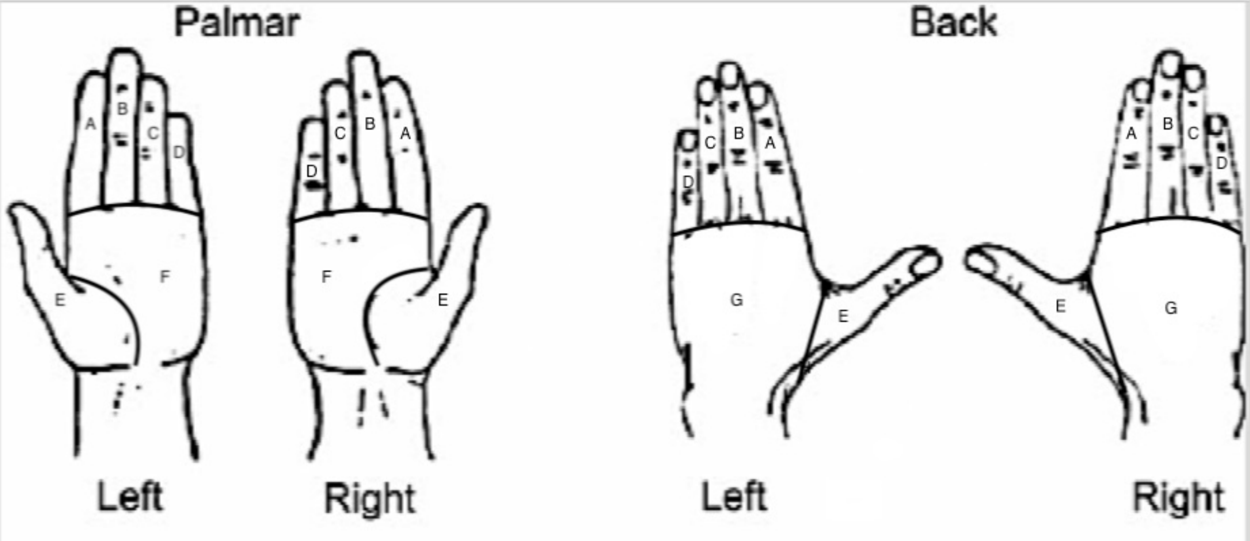

47. Using the letters on the above diagram, please indicate where and what type/s of symptoms have been felt *today*:
(More than one response per row can be selected if applicable)

	Pain	Discomfort	Stiffness	Aches	Pins and needles	Numbness
Left A - index finger	☐	☐	☐	☐	☐	☐
Left B - middle finger	☐	☐	☐	☐	☐	☐
Left C - ring finger	☐	☐	☐	☐	☐	☐
Left D - little finger	☐	☐	☐	☐	☐	☐
Left E - thumb	☐	☐	☐	☐	☐	☐
Left F - palm	☐	☐	☐	☐	☐	☐
Left G - back of hand	☐	☐	☐	☐	☐	☐
Left wrist	☐	☐	☐	☐	☐	☐
Right A - index finger	☐	☐	☐	☐	☐	☐
Right B - middle finger	☐	☐	☐	☐	☐	☐
Right C - ring finger	☐	☐	☐	☐	☐	☐
Right D - little finger	☐	☐	☐	☐	☐	☐
Right E - thumb	☐	☐	☐	☐	☐	☐
Right F - palm	☐	☐	☐	☐	☐	☐
Right G - back of hand	☐	☐	☐	☐	☐	☐
Right wrist	☐	☐	☐	☐	☐	☐

48. At the time of experiencing the above-mentioned symptoms, were you using the tablet device with one hand or two?

○ One hand only

○ Two hands

○ Alternating one hand at a time

Physical Impacts of Mobile Device Usage

Part 2: Body symptoms today - Tablet device

Body symptoms

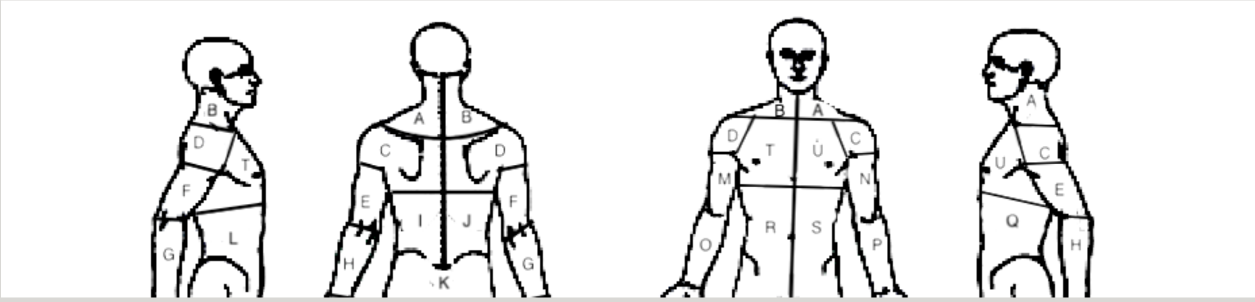

49. Using the letters on the above diagram, please indicate where and what type/s of symptoms have been felt *today*:
(More than one response per row may be selected if applicable)

	Pain	Discomfort	Stiffness	Aches	Pins and needles	Numbness
A - left side of neck	☐	☐	☐	☐	☐	☐
B - right side of neck	☐	☐	☐	☐	☐	☐
C - left shoulder	☐	☐	☐	☐	☐	☐
D - right shoulder	☐	☐	☐	☐	☐	☐
E - back of left arm	☐	☐	☐	☐	☐	☐
F - back of right arm	☐	☐	☐	☐	☐	☐
G - back of right forearm	☐	☐	☐	☐	☐	☐
H - back of left forearm	☐	☐	☐	☐	☐	☐
I - left back	☐	☐	☐	☐	☐	☐
J - right back	☐	☐	☐	☐	☐	☐
K - lower back	☐	☐	☐	☐	☐	☐
L - right side of body	☐	☐	☐	☐	☐	☐
M - front of right arm	☐	☐	☐	☐	☐	☐
N - front of left arm	☐	☐	☐	☐	☐	☐
O - palm side of right forearm	☐	☐	☐	☐	☐	☐
P - palm side of left forearm	☐	☐	☐	☐	☐	☐
Q - left side of body	☐	☐	☐	☐	☐	☐
R - right stomach	☐	☐	☐	☐	☐	☐
S - left stomach	☐	☐	☐	☐	☐	☐
T - right chest	☐	☐	☐	☐	☐	☐
U - left chest	☐	☐	☐	☐	☐	☐

Physical Impacts of Mobile Device Usage

Part 2: Symptoms and usage of tablet device

50. Please add any further information about symptoms you have felt today:

51. How long after starting to use your tablet device did you experience these symptoms?

- 0 - 15 minutes
- 15 - 30 minutes
- 30 - 60 minutes
- 1 - 2 hours
- 2 - 3 hours
- 3 - 4 hours
- 4 - 5 hours
- More than 5 hours

52. What position/s were you mainly in while using your tablet device?

- Sitting
- Standing
- Lying on back
- Lying on side
- Lying on front
- Moving around/varying position
- Other (please specify)

53. How would you rate the worst pain you have experienced, during or after using your tablet device *today*?

No pain | Moderate pain | Unbearable pain

Physical Impacts of Mobile Device Usage

End of Part 2

Thank you for entering your information into this survey.

If this was your first entry into Part 2, please access this survey once more within the 2 week survey period.

If this was your second entry into Part 2, thank you very much for completing all stages of this research project.

Your time and input are gratefully acknowledged and appreciated.
